# Infection of *Tilapia tilapinevirus* in Mozambique Tilapia (*Oreochromis mossambicus*), a Globally Vulnerable Fish Species

**DOI:** 10.3390/v13061104

**Published:** 2021-06-09

**Authors:** Pitchaporn Waiyamitra, Chutchai Piewbang, Somporn Techangamsuwan, Woei Chang Liew, Win Surachetpong

**Affiliations:** 1 Department of Veterinary Microbiology and Immunology, Faculty of Veterinary Medicine, Kasetsart University, Bangkok 10900, Thailand; w.doublep@gmail.com; 2 Department of Pathology, Faculty of Veterinary Science, Chulalongkorn University, Bangkok 10330, Thailand; alkaline_eart@hotmail.com (C.P.); somporn62@hotmail.com (S.T.); 3 Animal Virome and Diagnostic Development Research Group, Faculty of Veterinary Science, Chulalongkorn University, Bangkok 10330, Thailand; 4 Temasek Life Sciences Laboratory, Singapore 117604, Singapore; wcliew@gmail.com

**Keywords:** *Tilapia tilapinevirus*, TiLV, Mozambique tilapia, infection, pathology

## Abstract

*Tilapia tilapinevirus,* or tilapia lake virus (TiLV), is a highly contagious virus found in tilapia and its hybrid species that has been reported worldwide, including in Asia, the Americas, and Africa. In this study, we experimentally challenged Mozambique tilapia (*Oreochromis mossambicus*) with a virulent TiLV strain, VETKU-TV01, at both low (1 × 10^3^ TCID_50_/mL) and high (1 × 10^5^ TCID_50_/mL) concentration. After the challenge, the Mozambique tilapia showed pale skin with some hemorrhage and erosion, lethargy, abdominal swelling, congestion around the eye, and exophthalmos; there was a cumulative mortality rate at 48.89% and 77.78% in the groups that received the low and high concentration, respectively. Quantitative PCR and in situ hybridization confirmed the presence of TiLV in the internal organs of moribund fish. Notably, severe histopathological changes, including glycogen depletion, syncytial hepatic cells containing multiple nuclei and intracytoplasmic inclusion bodies, and infiltration of melanomacrophage into the spleen, were frequently found in the Mozambique tilapia challenged with high TiLV concentration. Comparatively, the infectivity and pathology of the TiLV infection in Mozambique tilapia and red hybrid tilapia (*Oreochromis* spp.) were found to be similar. Our results confirmed the susceptibility of Mozambique tilapia, which has recently been determined to be a vulnerable species, to TiLV infection, expanding knowledge that the virus can cause disease in this fish species.

## 1. Introduction

Tilapia Lake Virus Disease (TiLVD) is a new disease caused by *Tilapia tilapinevirus,* or tilapia lake virus (TiLV), which is a linear, negative-sense single-strand RNA virus containing ten segments; it has a total genome size of 10,323 kb [1,2]. Since 2014, TiLV has been reported on four continents, North America, South America, Asia, and Africa [2,3,4]. All stages of tilapia, from fry to adult and broodstock, are susceptible to TiLV, with mortality ranging from 5% to 100% [2,4,5,6,7,8,9]. TiLVD has been most extensively reported and studied in several species of tilapia, including Nile tilapia (*Oreochromis niloticus*) [5,8,10,11,12,13,14], hybrid tilapia (*Oreochromis niloticus* × *O. aureus*) [4], gray tilapia (*O. niloticus* × *O. aureus*) [15], and red hybrid tilapia (*Oreochromis* sp.) [8,16]. Some other species of fish are also susceptible to TiLV, including giant gourami (*Osphronemus goramy*), which showed a high mortality rate during laboratory challenge [17]; the virus was also detected in wild river barb (*Barbonymus schwanenfeldi*) in Malaysia [18]. TiLV primarily affects tilapia and its hybrid species, while most other freshwater fish are resilient to TiLV infection, including snakeskin gourami (*Trichogaster pectoralis*), iridescent shark (*Pangasianodon hypophtthalmus*), walking catfish (*Clarias macrocephalus*), striped snake-head fish (*Channa striata*), climbing perch (*Anabas testudineus*), common carp (*Cyprinus carpio*), silver barb (*Barbodes gonionotus*), Asian sea bass (*Lates calcarifer*) [17], and Indian major carp (*Labeo rohita*) [19].

Tilapias of the genus *Oreochromis* are a popular species for aquaculture in several regions of the world; three of the most common species are Nile tilapia (*Oreochromis niloticus*), blue tilapia (*Oreochromis aureus*), and Mozambique tilapia (*Oreochromis mossambicus*) [20,21,22,23,24]. Mozambique tilapia is known to grow more slowly, mature at a young age, and be more tolerant to wide salinity fluctuations than Nile tilapia [21,22,23,24,25]. Therefore, Mozambique tilapia has been used in breeding to improve the saltwater and cold tolerance of other tilapias [21,26,27]. The species has also been used as a model to study environmental factors [28,29] and response to pathogens [30]. Mozambique tilapia is native to the river of South Africa [31], but its numbers have decreased due to introduction of invasive fish species, including Nile tilapia. This invasion has resulted in the extirpation of Mozambique tilapia due to habitat competition and hybridization [32,33,34,35,36,37]. The Mozambique tilapia is now therefore considered a globally vulnerable species and is on the International Union for Conservation in Nature (IUCN) Red List [38]. In addition to habitat competition, pathogen carrying and spill over between native and invasive fish species will result in decreasing numbers of the native species. The susceptibility of Mozambique tilapia to pathogens that lead to massive decreases in number is therefore essential to investigate.

Although there is no direct experimental study on the susceptibility of Mozambique tilapia to TiLV, one previous study suggested that TiLV can replicate and infect their cells [39]. Furthermore, the natural infection of red hybrid tilapia with a Mozambique tilapia genetic background with TiLV (*O*. *niloticus* × *O. mossambicus*) has previously been reported [40]. Given the genetic similarities between Nile tilapia and Mozambique tilapia, there is a significant possibility that TiLV may cause infection in this species.

This study aimed to investigate the susceptibility of Mozambique tilapia (*O. mossambicus*) to TiLV infection by comparing TiLV infection in red hybrid and Mozambique tilapia, as well as comparing the infectivity and biology of the infection in Mozambique tilapia after exposure to both low and high concentrations of TiLV. It was determined that, regardless of the challenge dose, TiLV can cause clinical signs and mortality in Mozambique tilapia, though to different degrees of virus replication and severity.

## 2. Materials and Methods

### 2.1. Animals, Virus and Ethical Statement

Four hundred Mozambique tilapia (*Oreochromis mossambicus*) (average weight 15 g ± 0.5 g) were obtained from Temasek Life Sciences Laboratory, Singapore. The fish had no previous history of TiLV infection and were in good health post transportation to Thailand. Seventy red hybrid tilapias (average weight 10 g ± 0.5 g) were acquired from a tilapia hatchery with no history of TiLV infection from Saraburi province, Thailand. Both Mozambique tilapia and red hybrid tilapia were acclimatized at 28 °C in 400 L aquarium tank for seven days with daily water exchange at 50%. The water qualities were monitored every two days in terms of temperature (28–29 °C), pH (7.6–8.2), ammonia (0.2–0.5 ppm), and nitrite (0–0.03 ppm). Fish were fed with a commercial tilapia diet at the rate of 3% of total body weight per day. Before the experiment, five Mozambique tilapia and five red hybrid tilapia fish were randomly collected to screen for external parasites via skin scraping, gills biopsy, and bacterial colony count using tryptic soy agar. As previously described, a pool of spleen from five fish was processed for RNA extraction and TiLV screening by reverse transcription quantitative polymerase chain reaction (RT-qPCR) [41]. The animal use protocol was approved by the Institutional Animal Care and Use Committee of Kasetsart University, Bangkok, Thailand, under the protocol number ACKU61-VET-013. TiLV strain VETKU-TV01 was isolated in 2016 from red hybrid tilapia in a tilapia farm, Pathum Thani province, Thailand [42]. The virus was propagated in E-11 cells, a clone of SSN-1 cells [43]. The amount of TiLV (TCID_50_) was determined using Reed and Muench method [44].

### 2.2. Challenge Study

Three hundred and sixty Mozambique tilapia (weight 15 ± 0.5 g) were separated into three groups (four replicates/group) in a total of 12 tanks, with 30 fish/tank. Three tanks were used to record mortality and monitor morbidity and the fourth was used for sample collection. The details of each group are as follows: (1) control group; fish were intraperitoneally (IP) injected with uninfected E-11 cells and L-15 medium at 50 µL/fish; (2) low TiLV concentration; fish were IP-injected with TiLV at 1 × 10^3^ TCID_50_/mL, 50 µL/fish; (3) high TiLV concentration; fish were IP-injected with TiLV at 1 × 10^5^ TCID_50_/mL, 50 µL/fish. The sixty red hybrid tilapia were equally divided into two tanks, a sampling tank and a mortality recording tank. All red hybrid tilapia were IP-injected with high TiLV concentration at 1 × 10^5^ TCID_50_/mL, 50 µL/fish. At 0, 3, 6, and 12 days post-challenge (dpc), five fish were randomly collected from a sampling tank and processed for RT-qPCR and histopathology. Five red hybrid tilapia (control fish) were IP-injected with uninfected E-11 cells and L-15 medium at 50 µL/fish. All fish were euthanized by an overdose of eugenol (Aquanes^®^; Better Pharma; Bangkok, Thailand). The spleens were collected and stored at −20 °C until processed.

### 2.3. RNA Extraction and cDNA Synthesis

Total RNA was extracted from the spleens (approximately 30 mg of tissue) of both the Mozambique and red hybrid tilapia at the defined experimental time points using 1 mL of TRIzol^®^ reagent (Invitrogen, Carlsbad, CA, USA) according to the manufacturer’s instructions. Total RNA concentration and quality were estimated using spectrophotometry (NanoDrop^™^ 2000; Thermo Fisher Scientific Inc.; Waltham, MA, USA). For cDNA synthesis, 1 µg of total RNA was converted to cDNA using ReverTraAce^®^ qPCR RT kit (TOYOBO CO., LTD., Osaka, Japan) according to the manufacturer’s protocol. The cDNA templates were stored at −80 °C until analysis.

### 2.4. Quantitative Polymerase Chain Reaction (qPCR)

The qPCR was analyzed in a CFX96 real-time PCR thermocycler (Bio-Rad, Hercules, CA, USA) using an SYBR-based qPCR assay [41]. Briefly, the reaction consisted of 5 µL of 2 × iTaq™ universal SYBR Supermix (Bio-Rad, Hercules, CA, USA), the 0.3 µL forward primer was TiLV-112F 5′-CTGAGCTAAAGAGGCAATATGGATT-3′, and the 0.3 µL reverse primer was TiLV-112-R 5′-CGTGCGTACTCGTTCAGTATAAGTTCT-3′, 200 ng of cDNA template and molecular water to an absolute volume of 10 µL. The cycling condition was as follows: 95 °C for 3 min, 40 cycles of denaturing at 95 °C for 10 s, annealing and extension at 60 °C for 30 s. At the end of the qPCR reaction, melting curve analysis was carried out at 65 °C to 95 °C with 0.5 °C per 5 s increment. The calculation of the amount of TiLV genomic RNA in the spleen tissue was performed by SYBR-based qPCR assay as previously described [41]. The amount of TiLV genomic RNA was extrapolated from Ct value of each sample by comparing it to the standard curve of ten-fold serial dilutions of plasmid containing TiLV segment 3, as previously described [45].

### 2.5. Histopathology

The liver and spleen samples from the control and challenge groups were fixed in 10% (*v*/*v*) formalin for 24 h and then transferred into 70% ethanol solution. The samples were embedded in paraffin wax, sectioned, and stained with Hematoxylin and Eosin (H&E). The slides were scanned using VS120^®^ Virtual Microscopy Slide Scanning (Olympus, Tokyo, Japan) and examined under an Olympus OlyVIA Ver.3.1 program (Olympus, Tokyo, Japan).

### 2.6. In Situ Hybridization

The in situ hybridization (ISH) protocol was performed in 4 µm thick formalin-fixed paraffin-embedded (FFPE) slides according to the previously described protocol [46]. Briefly, the TiLV probe covering 491 bp of segment 3 of TiLV was amplified using the digoxigenin (DIG)-labeled oligonucleotides and prepared using a PCR DIG Probe Synthesis Kit (Roche Diagnostics, Basel, Switzerland). The FFPE slides were deparaffinized, rehydrated, and then rinsed in phosphate-buffered saline (PBS) pH 7.4. Subsequently, the slides were digested with Proteinase K, post-fixation, acetylation, and pre-hybridization, then hybridized with the 20 ng/µL specific probes at 42 °C overnight. Thereafter, the DIG-labelled signals were visualized using 100 µL of anti-DIG-alkaline phosphatase Fab fragments antibody (Roche, Basel, Switzerland) (1:200 in 1× blocking solution) in a combination of the StayGreen/AP Plus (Abcam, Cambridge, MA, USA). The slides were then counterstained with Nuclear Fast Red. Sections were considered positive for TiLV infection if green precipitates in the association of cellular morphology were visualized. Non-specific staining was assessed using an irrelevant canine bocavirus-2 (CBoV-2) probe [47].

## 3. Results

### 3.1. Susceptibility of Mozambique Tilapia to Tilapia Lake Virus

**Figure 1:**
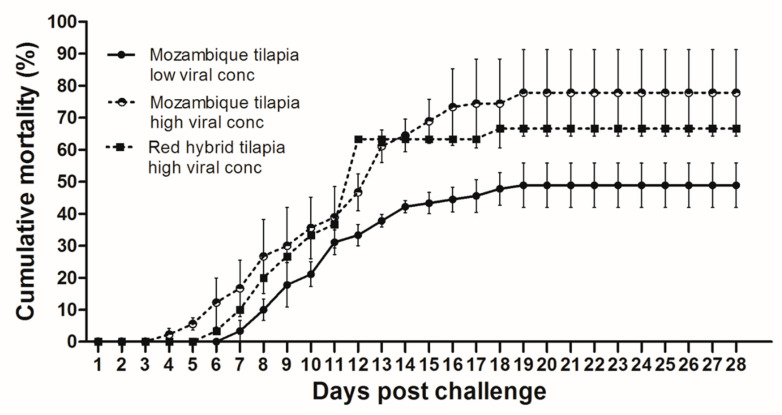
Cumulative mortality after Tilapia lake virus (TiLV) infection in Mozambique tilapia (*Oreochromis mossambicus*) and red hybrid tilapia (*Oreochromis* spp.). Fish were intraperitoneally challenged with TiLV at low viral concentration (1 × 10^3^ TCID_50_/mL) and high viral concentration (1 × 10^5^ TCID_50_/mL) per fish. Graph represents mortality in each group with triplicates, 30 fish per tank (a total of 90 fish/group). Mortality was recorded for 28 days.

**Figure 2:**
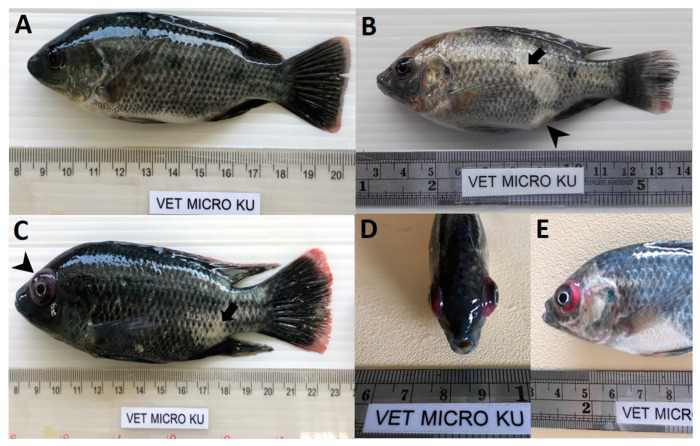
Clinical signs of TiLV infection in Mozambique tilapia (*Oreochromis mossambicus*). (**A**) Control uninfected fish. (**B**) Fish infected with TiLV at low viral concentration (1 × 10^3^ TCID_50_/mL), skin erosion (arrow) and abdominal swelling (arrowhead). (**C**) Fish infected with TiLV at high viral concentration (1 × 10^5^ TCID_50_/mL), skin erosion (arrow) and exophthalmos (arrowhead). (**D**,**E**) Fish infected with TiLV at high viral concentration (1 × 10^5^ TCID_50_/mL), exophthalmos and congestion of the eye.

At 28 dpc, the cumulative mortality in Mozambique tilapia with low (1 × 10^3^ TCID_50_/mL) and high (1 × 10^5^ TCID_50_/mL) concentration of TiLV was 48.89% and 77.78%, respectively (Figure 1). At 4 dpc, moribund Mozambique tilapia developed clinical signs of TiLV infection, including skin hemorrhage, lethargy, pale skin, abdominal swelling, skin erosion, congestion around the eye, and exophthalmos (Figure 2). Notably, mortality in fish exposed to high TiLV concentration began at 4 dpc, while the mortality in fish exposed to low TiLV concentration did not start until 7 dpc. Mortality in both low TiLV and high TiLV-exposed fish ended at 19 dpc (Figure 1). Intraperitoneal challenges (IP) of red hybrid tilapia with TiLV at 1 × 10^5^ TCID_50_/mL were conducted to compare clinical signs, pathology, and mortality patterns. Similar to the Mozambique tilapia, the red hybrid tilapia also showed skin hemorrhage, swimming at the bottom of the tank, skin and fin erosion, exophthalmos, and scale protrusion, with the first mortality at 6 dpc. At the end of the experiment, the cumulative mortality of the red hybrid tilapia was 66.67% (Figure 1). No clinical signs and mortality developed in the control Mozambique and red hybrid tilapia.

### 3.2. Confirmation of TiLV Infection in Moribund Mozambique Tilapia

To further confirm the susceptibility of Mozambique tilapia to TiLV, spleens were collected from low and high TiLV-challenged fish at 3, 6, and 12 dpc. As shown in Table 1, TiLV was detected in 3 out of 5 fish (60%) and 4 out of 5 fish (80%) exposed to the low concentration of TiLV at 3 and 6 dpc, respectively, with the virus concentration ranging from 9.7 × 10–4.8 × 10^5^ copies per μg of total RNA. No TiLV was detected in the fish exposed to the low concentration at 12 dpc. Conversely, all of the fish exposed to high concentration of TiLV had TiLV in their spleen as early as 3 dpc (1.1 × 10^4^–3.5 × 10^4^ copies per μg of total RNA), which remained detectable in all fish until 12 dpc (3.3 × 10^5^–2.3 × 10^6^ copies per μg of total RNA). Throughout the studied period, no TiLV was detected in the unchallenged fish.

### 3.3. Histopathological Finding and In Situ Hybridization in the Challenge Fish

**Figure 3:**
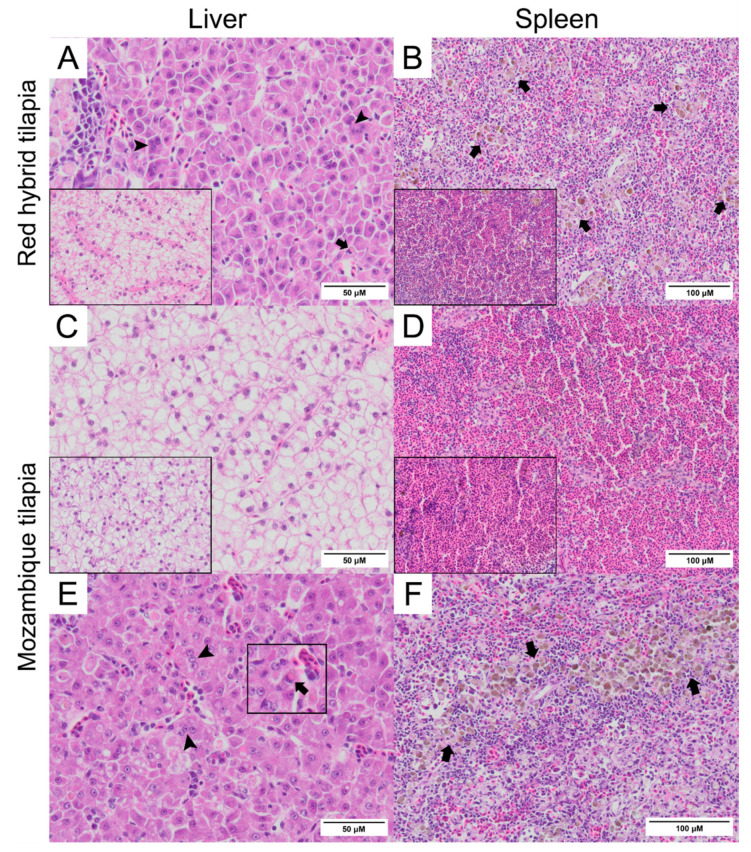
Histopathology of liver and spleen of red hybrid tilapia (*Oreochromis* spp.) and Mozambique tilapia (*Oreochromis mossambicus*) after 6 days post TiLV challenged. (**A**) Liver of red hybrid tilapia challenged with high TiLV concentration; depletion of glycogen, syncytial cells formation (arrowheads) and intracytoplasmic inclusion bodies (arrows), normal liver (inlet). (**B**) Spleen of red hybrid tilapia challenged with high TiLV showed depletion of red blood cells and increased melanomacrophage centers (MMCs) (arrows), normal spleen of red hybrid tilapia (inlet). (**C**) Liver of Mozambique tilapia challenged with low TiLV concentration, normal liver of Mozambique tilapia (inlet). (**D**) Spleen of Mozambique tilapia challenged with low TiLV concentration, normal spleen of Mozambique tilapia (inlet). (**E**) Liver of Mozambique tilapia challenged with high TiLV concentration; glycogen depletion, syncytial giant cells contained multiple nuclei (arrowheads) and intracytoplasmic inclusion bodies (inlet, arrows). (**F**) Spleen of Mozambique tilapia challenged with high TiLV concentration, infiltration of MMCs (arrows).

**Figure 4:**
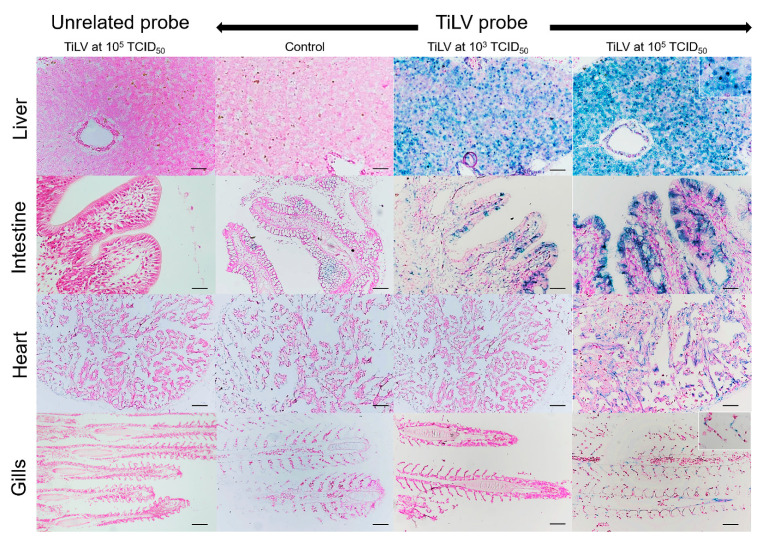
In situ hybridization (ISH) of TiLV RNA detection in Mozambique tilapia inoculation of TiLV at 1 × 10^3^ TCID_50_/mL and 1 × 10^5^ TCID_50_/mL concentrations. The TiLV RNA was detected in liver and intestinal epithelium of TiLV-infected Mozambique tilapia in both inoculation concentrations. The TiLV RNA labelling was evidence in the nucleus of hepatocytes (above inset), intestinal epithelium, myocardium, and gills epithelium (lower inset) of experimental Mozambique tilapia inoculation with TiLV at 1 × 10^5^ TCID_50_/mL concentration. No evidence of hybridization signal was observed in the control fish and in the TiLV-infected Mozambique tilapia sections inoculation with CBoV-2 probe (unrelated probe). Bars indicate 120 µm.

Normal hepatocytes with adequate glycogen storage were found in the liver of control fish (Figure 3A,C). No extensive histopathological changes were found in the livers and spleens of Mozambique tilapia that were challenged by the low concentration of TiLV (Figure 3C,D). However, severe histopathological changes could be found in red hybrid tilapia and Mozambique tilapia exposed to high concentration of TiLV at 6 dpc. Depletion of glycogen storage, loss of sinusoid structure, presence of syncytial giant cells containing multiple nuclei, and intracytoplasmic inclusion bodies were observed in the livers of both species (Figure 3A,E). A few melanomacrophage centers (MMCs) and normal lymphocytes surrounded by a sheath of reticular cells were observed in the spleen of the control red hybrid and Mozambique tilapia (Figure 3B,D), while an abundance of MMCs and depletion of red blood cells were observed in the spleens of the fish exposed to the high TiLV concentration (Figure 3B,F). No remarkable change was observed in the gills or intestines of the challenged fish. The localization of TiLV RNA in infected tissues including liver, intestines, heart, and gills was further confirmed by ISH. Intriguingly, positive signals with green coloration were clearly observed in all tested Mozambique tilapia tissues exposed to the high TiLV concentration, while positive signals were noted to a lesser extent in liver and intestines of Mozambique tilapia exposed to the low TiLV concentration. Conversely, no positive hybridization signal was detected in either the infected tissues exposed to a non-irrelevant probe or the uninfected control tissue samples (Figure 4).

## 4. Discussions

*Tilapia tilapinevirus*, aka tilapia lake virus (TiLV), was first described in farmed and wild tilapia in Israel [4] and later reported in sixteen additional countries [2,48,49]. Although the potency of TiLV has been demonstrated in various species of tilapia including Nile tilapia (*Oreochromis niloticus*), blue tilapia (*O. aureus*), and its hybrid species [4,5,15,42,50,51], there have been no reports of TiLV infection in Mozambique tilapia, which is genetically close to the Nile tilapia, a susceptible host to TiLV infection. This study showed that Mozambique tilapia (*O. mossambicus*) is also susceptible to TiLV infection. Generally, high morbidity and mortality occurred within ten days post challenge. Moribund fish showed pale skin with occasional hemorrhage and erosion, lethargy, abdominal swelling, congestion around the eye, and exophthalmos. Notably, the cumulative mortality in the groups that received low and high TiLV concentration was 48.89% and 77.78%, respectively. This high mortality in Mozambique tilapia is similar to previous reports in red hybrid (*Oreochromis* spp.) and Nile tilapia (*O. niloticus*), with cumulative mortality in these species ranging from 63–86% [17,42]. Mugimba et al. (2019) [15] reported 80–100% mortality in gray tilapia (*O. niloticus* × *O. aureus*) and red tilapia (*Oreochromis* spp.) after experimental challenge by TiLV.

Although the IP challenge did not mimic the natural route of virus infection as compared to the cohabitation challenge model, it provides more benefits, including an equal challenge dose in each individual fish. It also makes it more practical to compare changes in genes in the population, and most of the laboratory-based infection experiments rely on the IP injection of the virus infection [4,15,42,52]. Certainly, the strain of the virus, challenge dose, and species of tilapia could affect the infection’s outcome and lead to different mortality rates in these studies. The clinical signs in the observed Mozambique and red hybrid tilapia were quite similar to the clinical signs reported in other tilapias [5,12,42,50,53]. Additionally, the histopathological changes in TiLV-infected Mozambique tilapia include syncytial cell formation and degeneration of hepatocytes, depletion of red blood cells and increased MMCs in the spleen. Expectedly, infection with high TiLV concentration induces severe histopathological changes and the presence of positive signal in different organs of the fish. Notably, distinct histopathological changes, including glycogen depletion, syncytial giant cells contained multiple nuclei, and intracytoplasmic inclusion bodies were found in the liver; depletion of red blood cells and increased MMCs in the spleen of Mozambique tilapia and red hybrid tilapia received high TiLV concentration. Comparatively, these pathological changes were reported in different species of fish under natural and experimental challenge by TiLV [42,46,54,55].

Previously, TiLV-genomic RNA was detected in the intestine of Nile tilapia as early as 24 h after intragastric challenge [54]. The results of this study revealed a positive hybridization of TiLV in multiple organs, including the intestine, gills, heart, and liver, of TiLV-challenged Mozambique tilapia tissues. Interestingly, a negative ISH was found in heart and gills of the Mozambique tilapia that received the low TiLV concentration. Such distinct ISH reaction in organs based on the TiLV concentration given to the fish suggests virus tropisms in the tissues of the fish. A previous study found that many types of tilapia tissue, such as the gills, brain, liver, and head kidneys, showed positive signals of ISH reaction [10,17]. As expected, high TiLV challenge led to severe histopathological alterations, which is in accordance with the presence of high TiLV RNA in tissues. This is the first study to report the susceptibility of Mozambique tilapia to TiLV; it is important to note that both red hybrid and Mozambique tilapia show similar patterns of morbidity and mortality after TiLV infection in that mortality begins at 4–6 days post challenge. Based on phylogenetic analysis and sequence comparisons among tilapia species, it has been suggested that these species have a close genetic background with possible similar target cells, receptors, or details to support TiLV replication [56,57,58]. Moreover, the presence of TiLV-genomic RNA was detected in the spleen of the Mozambique tilapia, confirming its susceptibility to TiLV. Indeed, TiLV persists in fish challenged with high TiLV concentration until 12 dpc, while no virus was detected in fish inoculated with low TiLV concentration at 12 dpc, suggesting that the immune system may contribute to the clearance of the virus at low concentration prior to establishment of effective infection. Given the detection of TiLV at 3 and 6 dpc in the low TiLV fish, we believe that the virus was successful in establishing the infection, but that the infectivity may not be efficient enough to overcome the immune response of the fish. A significant expression of immune-related genes including Mx was reported in zebrafish upon TiLV challenge [52]. Thus, the activation of an antiviral response occurs during early infection and could provide immediate protection. Previous studies demonstrated that Mozambique tilapia is susceptible to many pathogens, including virus [59] and bacteria [60,61]. Our study extends the current knowledge by showing that TiLV, one of the important emerging diseases in tilapia, can cause disease in the species. As the Mozambique tilapia is now on the IUCN Red List due to habitat competition from Nile tilapia, prevention of a common tilapia pathogen like the TiLV infection in natural Mozambique tilapia is necessary for the conservation of this species.

In conclusion, our results demonstrated that Mozambique tilapia is susceptible to TiLV infection. It remains unknown whether any underlying factors could contribute to difference in susceptibility of TiLV in different tilapia strains. Nonetheless, the study confirmed that TiLV can cause TiLVD in the Mozambique tilapia, with clinical signs and pathology similar to those found in the Nile and red hybrid tilapia. This information could be considered in aquaculture practice, as many fish species share water resources and are commonly cultured in the polyculture system. As such, avoiding the introduction of susceptible fish species and the implementation of appropriate controls will reduce the risk of introducing TiLV to both farms and nature.

## Figures and Tables

**Table 1:** Persistence of TiLV genomic RNA in spleen of challenged Mozambique tilapia.

Positive Samples/Total Sample			
(Number of Viral Copies/µg of Total RNA)			
Day Post Challenge (DPC)	Control	Low TiLV Conc.	High TiLV Conc.
		(1 × 10^3^ TCID_50_/mL)	(1 × 10^5^ TCID_50_/mL)
3	0/5	3/5	5/5
		(9.7 × 10^–1^ × 10^3^)	(1.1 × 10^4^–3.5 × 10^4^)
6	0/5	4/5	5/5
		(1.3 × 10^3^–4.8 × 10^5^)	(2.7 × 10^2^–2.6 × 10^6^)
12	0/5	0/5	5/5
			(3.3 × 10^5^–2.3 × 10^6^)

## Data Availability

The data presented in this study are available on request from the corresponding author.
